# Use of AI in Diagnostic Imaging and Future Prospects

**DOI:** 10.31662/jmaj.2024-0169

**Published:** 2024-10-08

**Authors:** Norikatsu Miyoshi

**Affiliations:** 1 Department of Gastroenterological Surgery, Osaka University, Suita, Japan; 2 Department of Innovative Oncology Research and Regenerative Medicine, Osaka International Cancer Institute, Osaka, Japan

**Keywords:** Diagnostic Imaging, AI, Artificial Intelligence, Gastroenterological Surgery

## Abstract

**Introduction:**

The integration of artificial intelligence (AI) into medical practices has transformed fields like gastroenterological surgery. AI predicts patient prognoses using clinical and pathological data and develops technologies that create three-dimensional (3D) models for surgical simulations, thereby enhancing surgical precision and care quality.

**Methods:**

At our facility, AI-driven diagnostic and treatment systems have been developed under the “Strategic Innovation Creation Program” by the Cabinet Office. Our research focuses on perioperative care by constructing 3D models from preoperative imaging data to develop surgical support systems for preoperative simulations and navigation during surgery. Additionally, we use deep learning to predict disease progression and complications and natural language processing to analyze electronic medical records to predict postoperative complications.

**Results:**

AI-based surgical support systems effectively convert two-dimensional imaging data into 3D models, thereby improving surgical precision. Predictive models for disease progression and complications developed using deep learning have high accuracy. AI applications in diagnostic imaging enable early detection and improved treatment planning. AI-based tools for informed consent and patient support enhance patient understanding and satisfaction.

**Conclusions:**

AI revolutionizes medical practices by improving diagnostic accuracy, surgical precision, and patient outcomes. Future projects will integrate remote diagnostic and treatment planning; leverage AI for comprehensive, high-quality care; and support work-style reforms for healthcare professionals. Advancements in AI will overcome current medical challenges and enhance the communication between physicians and patients.

## Introduction

Recent advances in artificial intelligence (AI) have exerted a profound effect on our daily lives, ranging from speech and image recognition to translation applications. The field of medicine has also experienced an effect as AI technologies are integrated into various aspects of healthcare. In gastroenterological surgery, clinical and pathological data, such as patient height, weight, medical history, blood test results, and imaging data (such as computed tomography (CT) scans), are extensively used to predict individual patient prognoses. Although computer algorithms have traditionally been used in this analysis, AI is increasingly being applied in research and analysis in this field.

At our facility, aiming to realize Society 5.0, we have been developing advanced diagnostic and treatment systems using AI in hospitals under the “Strategic Innovation Creation Program” by the Cabinet Office. Expanding on this research, our focus has been on implementing AI in perioperative care within the field of gastroenterological surgery. This includes analyzing electronic medical record data, applying diagnostic imaging techniques, and using AI to assist patient understanding. In particular, we have focused on constructing three-dimensional (3D) models based on preoperative imaging data obtained by endoscopy, CT, and magnetic resonance imaging (MRI). These models allowed us to develop surgical support systems that simulate preoperative scenarios and share information during surgery. These systems are crucial for team-based surgical procedures in which sharing information about the anatomical location of target organs, diseases, and appropriate resection ranges is vital.

## AI-based Surgical Support Systems

Transforming two-dimensional (2D) imaging data from clinical scans (e.g., CT and MRI) into 3D models is made possible using medical devices. By assigning labels to the 2D data of body surfaces and internal organs (such as the viscera, blood vessels, muscles, and bones), we can construct 3D models with a single click. This process facilitates postoperative functional evaluation and reconstruction planning by measuring the volume of resected organs, which is a crucial function that is extensively used in gastroenterological surgery.

**Figure 1.**
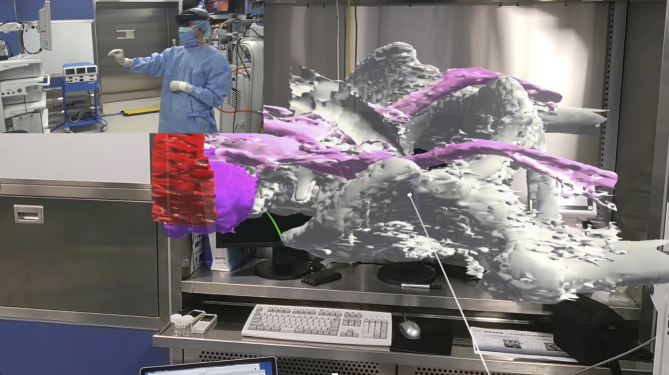
Projection of a three-dimensional (3D) model into space to illustrate the manipulation of the 3D model during surgery. This figure demonstrates the intuitive display of cross-sectional images of a 3D model in space without the need for special controllers.

We have been conducting research using clinical data for navigation surgery in the surgical field. By converting standard Digital Imaging and Communications in Medicine image data into polygon data (3D data) and outputting them in an editable format, we developed a system to project these models onto the surgical field through projection mapping, displaying 3D models in physical space. This system allows real-time observation of arbitrary cross sections (Figure 1), enabling a detailed simulation of the surgical site preoperatively and intraoperartively. This approach aids in understanding the precise anatomical location of lesions and their spatial relationship with surrounding structures, especially in intricate pelvic surgeries.

**Figure 2.**
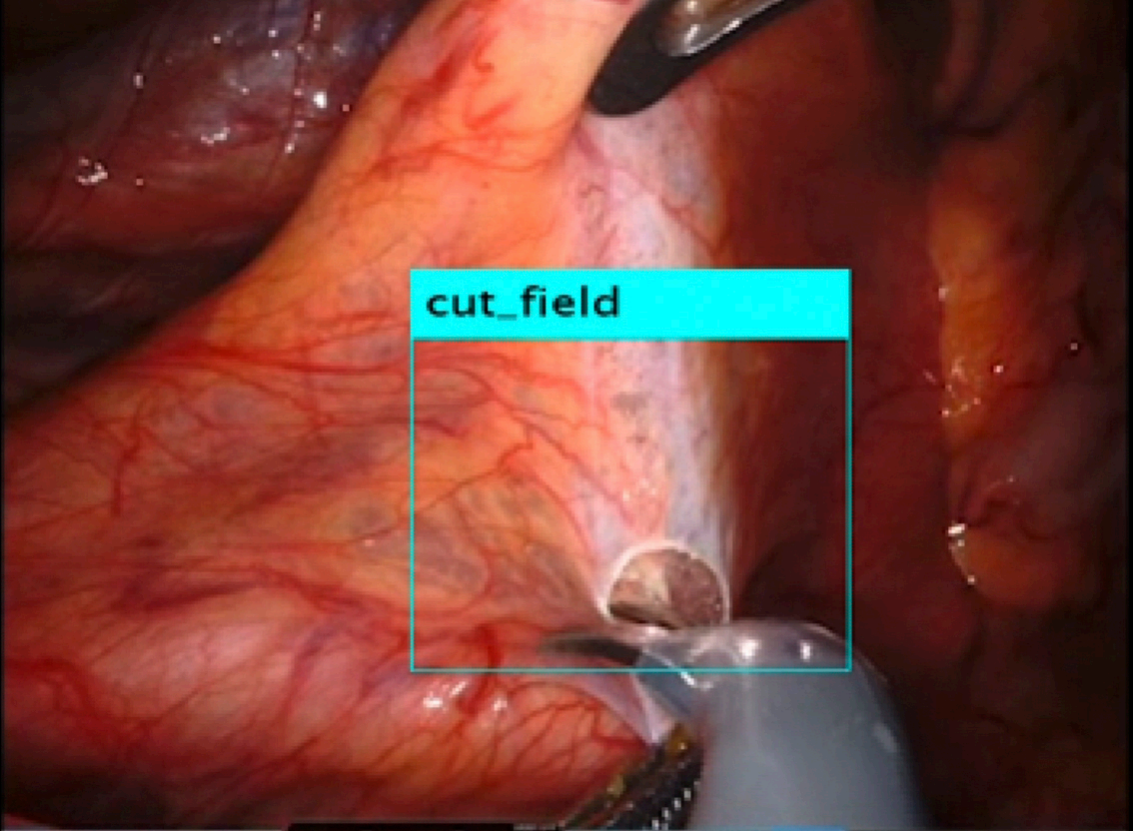
Artificial intelligence (AI) video analysis results for surgical navigation in robotic surgery. This figure shows the starting point of the incisions and areas supported by the robotic arm, resulting in standardized and safe surgery.

**Figure 3.**
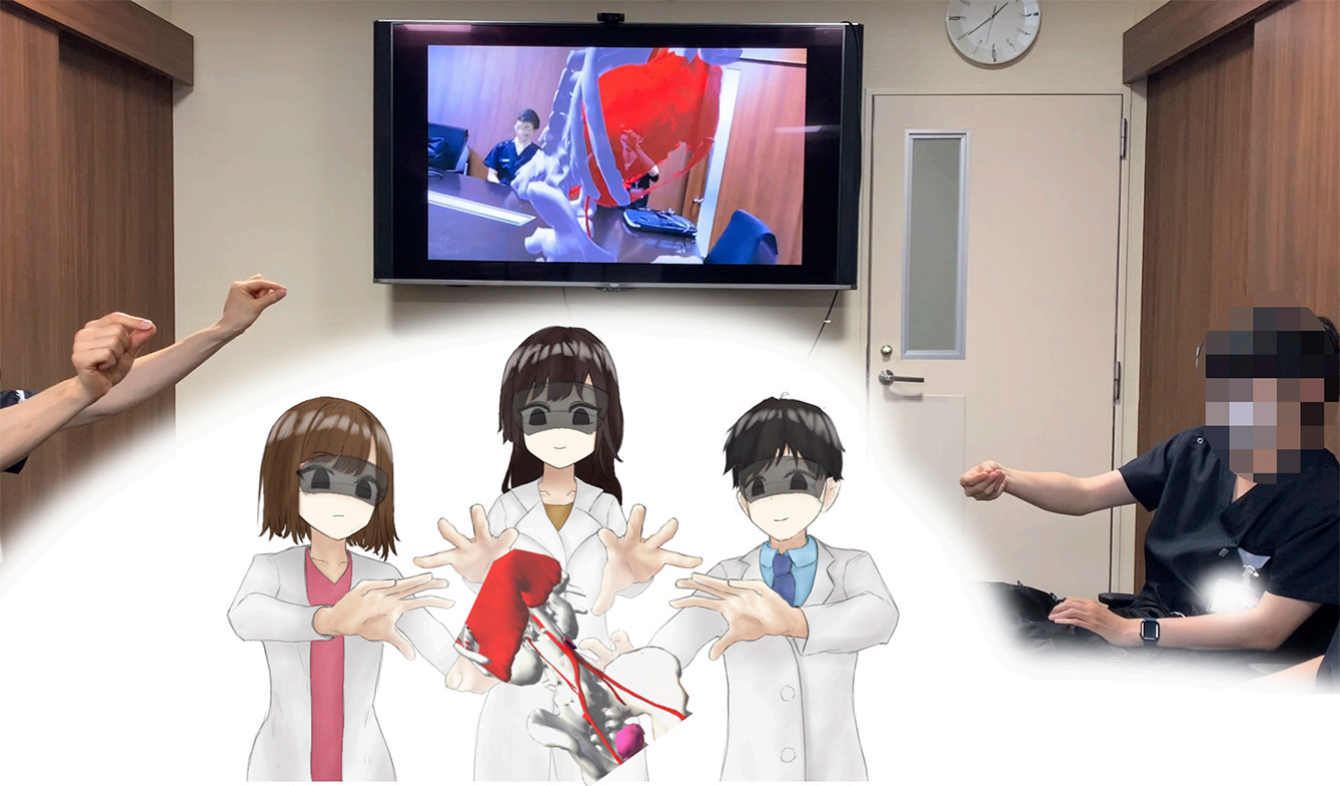
Application of immersive devices for surgical education, including three-dimensional (3D) models and virtual and augmented reality devices for educational purposes for students and young doctors.

Recently, the use of laparoscopy and robotic surgery has become widespread. These techniques make it challenging to palpate lesions directly, highlighting the importance of accurately identifying the location of lesions and effectively communicating this information with the surgical team. Our current research focused on exploring the use of video-surgery-specific image data for surgical navigation (Figure 2). By visualizing lesions previously “inferred” by surgeons from preoperative imaging data, we can create valuable tools for surgical simulations and educational purposes for students and young surgeons (Figure 3).

Additionally, we are currently investigating the use of wearable devices for surgical navigation, leveraging AI and communication technologies to support medical care in regions with a shortage of specialists.

## Predicting Disease Progression and Complications Using AI

Gastrointestinal endoscopy allows for a detailed diagnosis and, in certain cases, lesion treatment. Observing lesions on the luminal surface allows clinicians to judge malignancy and progression, which is crucial for treatment planning. However, accurately diagnosing the depth of lesions using only endoscopy can be challenging and often requires additional tests, such as ultrasound, CT, and MRI.

At our facility, we are investigating the potential of AI to predict the depth of lesions from endoscopic images. By collecting and analyzing image data from past cases using deep learning in MATLAB ^(1)^, we predicted the depth of the lesions, especially the invasion depth. We use deep neural networks and sensitivity analysis techniques, including occlusion, to evaluate the effect of perturbations on input images, helping identify important regions for classification ^(1), (2)^.

**Figure 4.**
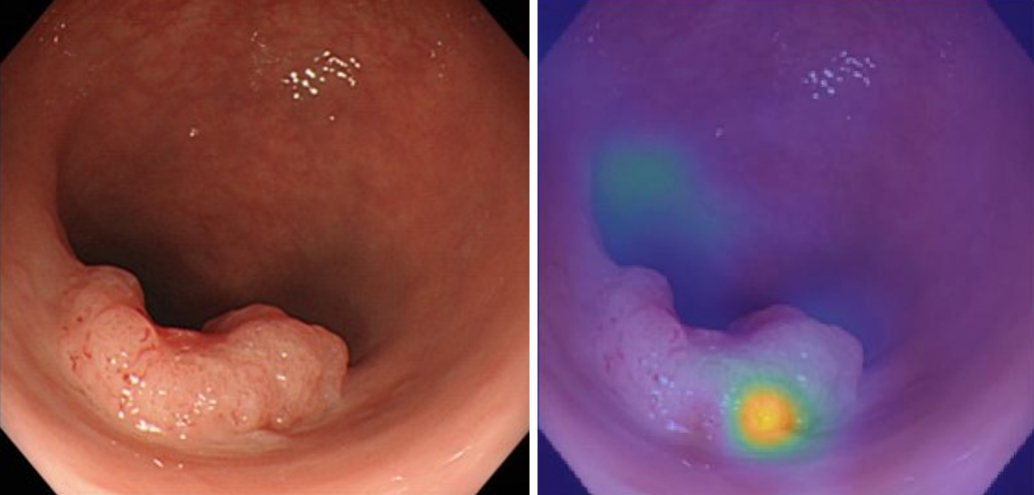
Artificial intelligence (AI) analysis of endoscopic images. (Left) Endoscopic image photo. (Right) AI analysis of lesion depth using a heat map indicating the regions assessed using AI.

We annotated endoscopic images of 177 colorectal cancer cases with postoperative pathological diagnoses and developed a program to predict “depth” using deep learning. This allowed us to identify cases in which endoscopic local resection alone was feasible and to develop a formula for this prediction (Figure 4) ^(3)^. By combining this information with other imaging data and test results, we can accurately evaluate cases with minimal invasiveness.

Furthermore, we are investigating AI applications to predict the “final treatment effect” during treatment progression ^(4)^. By annotating preoperative imaging data with clinical course data and using deep learning techniques, we can predict treatment outcomes and facilitate early adjustments to treatment plans.

**Figure 5.**
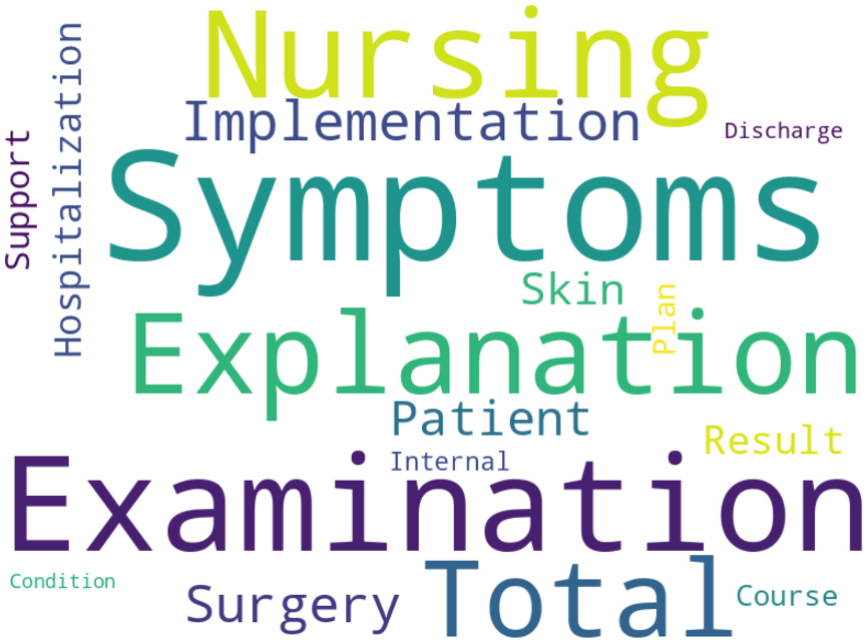
Artificial intelligence (AI) prediction of complications based on electronic medical records (EMRs), specifically forecasting the likelihood of postoperative fever through AI natural language analysis of EMRs. Word clouds indicate contributing keywords in the analysis (translated in English).

Additionally, we are exploring the use of AI for natural language processing of electronic medical record (EMR) data to predict postoperative complications (Figure 5). We developed a model using AI for natural language analysis to predict postoperative fever. Improving the accuracy of the model could facilitate early therapeutic interventions, etiological investigations, and ultimately reduce medical expenses.

## AI-based Medical Work and Patient Support Systems

Our research also delved into the realm of AI applications for informed consent (IC) in clinical settings, with the aim of improving patients’ and families’ understanding and satisfaction with IC explanations. These explanations typically encompass detailed explanations of the patient’s condition, treatment (surgery), postoperative course, complications, and sequelae. In collaboration with BIPROGY Inc. (Tokyo, Japan), we developed an application to analyze facial expressions, dynamic and pulse measurements, and voice data to estimate emotions during the IC process. This application enables a more personalized approach in tailoring responses to the understanding levels of patients and their families, facilitates data collection during IC sessions, and provides feedback for delivering more focused face-to-face explanations ^(5)^.

AI can be used to predict postoperative complications. For example, the use of AI to generate virtual images of potential future skin inflammation following stoma creation may facilitate early intervention and preventive measures. This approach can help improve patient outcomes and follow-up. Integrating AI technology with online consultations and advanced communication technologies can enhance patient understanding, alleviate anxiety, and provide personalized care, even in remote settings.

## Advancements in Diagnostic Imaging through AI

The potential of AI extends beyond surgical support and complication prediction. AI integration is revolutionizing diagnostic imaging. Advanced AI-based algorithms enable the analysis of imaging data that is beyond the capabilities of human interpretation. AI has the ability to detect patterns and anomalies in imaging data with unprecedented accuracy, thereby assisting radiologists in early diagnosis and treatment planning. AI-powered imaging tools can enhance the detection of subtle changes in tissue structure, thereby aiding in the early detection of diseases, including cancer. Additionally, these tools can predict tumor aggressiveness, thereby assisting in tailoring treatment plans for individual patients. The ability of AI to continuously learn and improve on new data ensures that diagnostic accuracy can evolve and become more refined over time ^(6), (7)^.

## AI in Radiology and Oncology

Radiology and oncology are fields in which AI has made significant progress. In radiology, AI algorithms can quickly analyze vast amounts of imaging data, identify abnormalities, and reduce the workload of radiologists. The accuracy of AI for detecting conditions such as breast cancer, presence of lung nodules, and brain tumors is continuously improving, leading to earlier and more accurate diagnoses ^(8)^.

## Role of AI in Personalized Medicine

Personalized medicine aims to tailor medical treatment according to individual characteristics of each patient. AI plays a crucial role in this approach by analyzing genetic, environmental, and lifestyle data to predict disease risk and treatment response. AI-driven models can identify the most effective interventions for each patient, improve outcomes, and reduce unnecessary treatment ^(9), (10)^. Genomic data analysis is a key area where AI is transforming personalized medicine. AI can aid in the development of targeted therapies by identifying genetic mutations associated with diseases. For instance, AI algorithms can analyze genomic data to identify patients who can benefit from specific cancer treatments, such as immunotherapy and targeted drug therapies ^(11)^.

## Challenges and Ethical Considerations

Although AI has great potential in the healthcare sector, it faces several challenges and ethical issues. Data privacy and security are of paramount concern, as AI relies on large datasets that include sensitive patient information. Ensuring that these data are safeguarded against breaches and misuse is crucial.

Moreover, the use of AI in decision-making raises ethical concerns regarding accountability and transparency. Ensuring that AI systems are transparent in their decision-making processes and that accountability exists for the outcomes of AI-driven decisions is imperative. Additionally, regulatory frameworks are necessary to ensure the safe and effective use of AI in health care. These frameworks should address issues such as the validation and certification of AI algorithms, the management of biases in AI systems, and the integration of AI into clinical workflows ^(12)^.

## Conclusions

Environmental improvements, such as work-style reforms for physicians, progress, and adaptation to new normal situations, including measures against COVID-19 and social distancing, are crucial. Providing better medical care while reducing the burden on patients and their families is of paramount importance.

In the future, our projects will realize future health care in which diagnoses and treatment plans can be made remotely based on existing data. By combining remote emotional and satisfaction estimation systems with necessary in-hospital tests and treatments, we provide comprehensive and high-quality medical care. Additionally, we strive to overcome current medical challenges in gastroenterological surgery using AI technology for diagnostics, treatments, and overall medical practice, thereby improving communication between physicians and patients and supporting work-style reforms.

## Article Information

### Conflicts of Interest

None

### Acknowledgement

I would like to thank the staff of the Department of Gastroenterological Surgery at Osaka University for their engagement and support during the trial. I also would like to express my gratitude to Ms. Misa Taguchi and Dr. Hiroyuki Hishida at The MathWorks, Inc. for their valuable discussions and the information provided regarding AI analysis.
